# The kidney–brain pathogenic axis in severe falciparum malaria

**DOI:** 10.1016/j.pt.2023.01.005

**Published:** 2023-02-02

**Authors:** Andrea L. Conroy, Dibyadyuti Datta, Angelika Hoffmann, Samuel C. Wassmer

**Affiliations:** 1 Department of Pediatrics, Indiana University School of Medicine, Indianapolis, IN 46202, USA; 2 University Institute of Diagnostic and Interventional Neuroradiology, University Hospital Bern, University of Bern, Bern, Switzerland; 3 Department of Infection Biology, Faculty of Infectious and Tropical Diseases, London School of Hygiene and Tropical Medicine, London, UK

## Abstract

Severe falciparum malaria is a medical emergency and a leading cause of death and neurodisability in endemic areas. Common complications include acute kidney injury (AKI) and cerebral malaria, and recent studies have suggested links between kidney and brain dysfunction in *Plasmodium falciparum* infection. Here, we review these new findings and present the hypothesis of a pivotal pathogenic crosstalk between the kidneys and the brain in severe falciparum malaria. We highlight the evidence of a role for distant organ involvement in the development of cerebral malaria and subsequent neurocognitive impairment post-recovery, describe the challenges associated with current diagnostic shortcomings for both AKI and brain involvement in severe falciparum malaria, and explore novel potential therapeutic strategies.

## An emerging link between kidneys and the brain in malaria

Despite recent efforts and successes in reducing the malaria burden globally, severe *P. falciparum* infection still accounted for the vast majority of the reported 619 000 malaria deaths in 2021 [1]. In addition to this staggering death toll, severe malaria is a leading cause of acquired **neurodisability** (see Glossary) in African children [2]. These long-term effects were long believed to be confined to patients recovering from cerebral malaria, a neurological syndrome characterized by unrousable coma and leading to a constellation of persistent neurological **sequelae** in up to 30% of pediatric survivors, which include cognitive impairment, motor skills, visual coordination, seizures and attention deficit hyperactivity disorder [2,3]. While these sequelae have been extensively investigated in pediatric patients, very few studies have been carried out in adults: they are thought to have fewer neurological sequelae than children, but well-designed cohort studies with serial follow-up assessments are currently lacking [4]. New evidence shows that the brain is also affected in patients without clinically evident neurological involvement at the time of infection, suggesting that the recorded neurodisabilities following cerebral malaria are only the tip of the iceberg. For example, adult respiratory distress syndrome, a common occurrence in severe malaria, can be associated with long-term impairment in cognitive function [5]. In children, complications including severe anemia [6] and **AKI** [7] have also been linked to cognitive impairment, leading to developmental delay years after recovery [8,9]. Among pediatric falciparum malaria patients enrolled in a clinical trial, neurologic deficits occurred in 5.4% of children without AKI compared to 33.3% in children with AKI [10]. In a large observational study of children with cerebral malaria, the frequency of neurologic deficits at discharge was 25.4% in children without AKI compared to 53.9% in children with AKI [7]. Overall, this indicates that neurocognitive sequelae following *P. falciparum* infection might be significant, with potentially important developmental and productivity consequences, as described in other conditions such as stroke [11].

In addition, several recent studies have highlighted a potential pivotal pathogenic crosstalk between the kidneys and the brain in severe falciparum malaria [7,8,12–17], not only demonstrating a new role for distant organ involvement in the development of neurocognitive sequelae post-recovery but also providing novel potential therapeutic strategies to prevent them in the future.

## Pathogenesis of severe *P. falciparum* infection and potential kidney–brain crosstalk pathways

**Figure 1.**
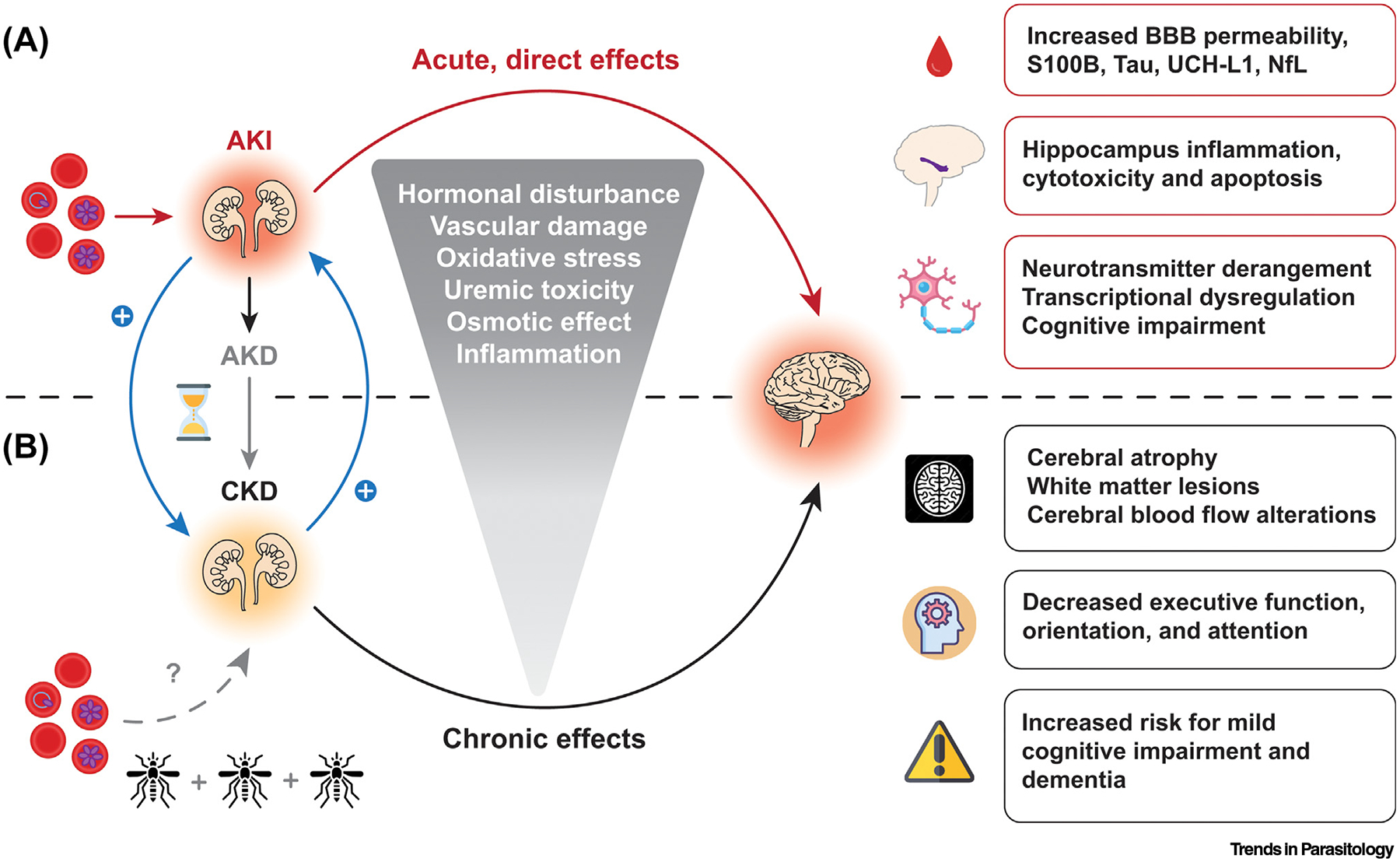
Proposed kidney–brain pathogenic axis in severe falciparum malaria. In addition to the direct effects of *Plasmodium falciparum*-infected erythrocytes on the brain during cerebral malaria, acute kidney injury (AKI) in severe malaria has been linked to blood–brain barrier (BBB) impairment, potentially leading to the diffusion of brain injury biomarkers into the bloodstream, to specific changes in the hippocampus, and to overall deranged physiological brain functions. (A) AKI can lead to a continuum of kidney disease with AKI representing an abrupt change in kidney function to acute kidney disease (AKD) characterized by impaired kidney function or structural injury <90 days, and chronic kidney disease (CKD) as kidney disease lasting more than 90 days. Repeated AKI episodes are a risk factor for CKD, and people living with CKD are also at risk for AKI (blue arrows). In turn, CKD has been associated with brain structure changes assessed through quantitative neuroimaging, brain function alterations, and increased risks for cognitive impairment and dementia. (B) There are currently no data available on the effects of repeated infections or chronic/asymptomatic malaria on the development of CKD (gray broken line, lower left quadrant). Abbreviation: NfL, neurofilament light chain.

The vascular **endothelium** is central to severe falciparummalaria pathogenesis. *P. falciparum* is an intracellular parasite that modifies the membrane of erythrocytes it invades, allowing them to **cytoadhere** to the endothelial cells to avoid splenic clearance. The resulting sequestration of parasitized erythrocytes in microvascular beds leads to impaired blood flow, a local inflammatory milieu, and endothelial activation that can result in tissue ischemia [18]. Microvascular obstruction, endothelial activation, and heme-mediated oxidative damage have been associated with the pathogenesis of cerebral malaria and malaria-associated AKI, indicating common pathophysiological mechanisms [19]. As end organs on parallel trajectories, the brain and kidneys have similar anatomical and functional vascular features, leading to a unique susceptibility to vascular injury. Kidney biopsies in severe malaria demonstrate accumulation of hemozoin-laden macrophages, vascular changes, including cortical necrosis, tubular necrosis, thrombotic microangiopathy, and reduced tight junctions in kidney tissue of patients with AKI, all supporting a central role for vasculopathy [20,21]. Cerebral malaria involves more dense parasite sequestration in the brain microvasculature, with mononuclear cell infiltrates, ring hemorrhages and loss of endothelial **glycocalyx** [22,23]. The kidney is a highly vascular organ involved in filtration of blood and is exceedingly susceptible to injury in the context of reduced blood flow due to hypovolemia, parasite sequestration, impaired autoregulation, and in conditions of elevated kidney toxins. Proximal tubular cells in the kidney are characterized by a high metabolic rate and low oxygen delivery from the capillaries. Consequently, the kidney is sensitive to vascular dysfunction, a phenomenon exacerbated by dehydration and hypovolemia, oxidative stress, and through exposure nephrotoxin medications [14,24–26]. In turn, through the release of inflammatory mediators, retention of uremic toxins, and hormonal disturbance, AKI can lead to **hippocampus** inflammation, cytotoxicity, and apoptosis, resulting in long-term cognitive impairment, as reviewed in [27,28] (Figure 1). Indeed, AKI has been associated with endothelial activation and loss of blood–brain barrier (BBB) integrity in inflammatory models such as sepsis, liver failure, and neurologic disease [29]. In severe falciparum malaria infection, kidney dysfunction can lead to systemic inflammation and endothelial activation, thereby contributing to BBB impairment [29,30]. Reduced kidney function results in the accumulation of several uremic solutes that can also impair normal brain function and are usually cleared by the kidneys. **Excitotoxic** metabolites elevated in cerebral malaria include quinolinic acid and kynurenic acid, both associated with endothelial activation and worse cognitive outcomes in pediatric malaria [16,31,32]. Hormonal disturbance following AKI include the activation of the renin–angiotensin–aldosterone axis and the subsequent increase in circulating angiotensin II, but also elevated plasma levels of natriuretic peptides and parathyroid hormone, which may all trigger brain inflammation and injury [27]. In addition, upregulation and accumulation of vasopressin in kidney failure could lead to posterior reversible encephalopathy syndrome (PRES) [33], a clinicoradiological entity recently described in patients with cerebral malaria [34]. While AKI may impact brain functions, the opposite has also been postulated. Brain injury, and in particular cerebrovascular diseases, could result in impaired renal function through both neuroendocrine and immune pathways [35]. These effects have not been investigated in malaria to date but may contribute to the high prevalence of AKI in adult [36] and pediatric cerebral malaria patients [7,13,14].

## New evidence for the role of AKI and chronic kidney disease in malaria-associated neurological sequelae

AKI is characterized by an abrupt loss of kidney function and should be defined using the Kidney Disease: Improving Global Outcomes (KDIGO) criteria based on a decrease in urine output or an increase in creatinine [37]. AKI also leads to a physiologic state of uremia characterized by a build-up of nitrogenous waste products with elevated blood urea nitrogen (BUN), the most well described marker associated with AKI in malaria. AKI and elevated BUN have recently been associated with the development of neurodisabilities in pediatric survivors from Uganda [7,13,14], with unresolved AKI following clinical stabilization (including fluid resuscitation) linked to the greatest risk [14]. Remarkably, children with severe malaria and AKI are at a higher risk for prolonged hospitalization, mortality, and chronic kidney disease (CKD). In a population of 368 Ugandan children with severe malaria, AKI on admission was associated with 2.81-fold increased odds of developing CKD at 1-year follow-up [7]. Given the burden of malaria in endemic areas and the risk of repeated AKI events over a child’s lifetime, this study provides an insight on how severe malaria during childhood may contribute to the widespread burden of CKD in adults. In addition to emerging data implicating AKI in worse cognition, CKD also commonly leads to cognitive impairment [38], even in children without established CKD risk factors (e.g., diabetes) [39,40]. Together, these findings suggest a feedback loop either exacerbating brain lesions in patients with neurocognitive sequelae or triggering them in those who were not affected during acute falciparum infection. Further deleterious effects through additive effects of subsequent infections during life are likely, and their impact in patients with ‘asymptomatic’ parasitemia is currently unknown. While the specific underlying mechanisms remain poorly understood, vascular damage, uremic toxicity, oxidative stress, changes in osmolarity, and peripheral/central inflammation induced by CKD are all suspected to be involved in brain changes assessed through brain function alterations, and cognitive decline [41–43].

## Cerebral malaria: a shifting definition

The World Health Organization (WHO)-defined criteria for cerebral malaria are used for the identification of patients needing more intensive care or to define patient groups for research [44]. These have broadened between 1999 and 2000, allowing the identification of a substantial additional number of cases with mild cerebral involvement [45]. Despite the hard cut-offs currently used to define the occurrence of coma, and therefore of cerebral malaria, the wide spectrum of neurological signs in severe malaria ranges from clinically evident to only apparent using neuroimaging modalities. This was recently demonstrated by a study in India using magnetic resonance imaging (MRI) in adults, which showed that the brain is commonly affected during severe but noncerebral malaria [17]. Indeed, reversible diffusion restriction in the basal ganglia was noted in approximately 20% of enrolled patients without cerebral involvement according to the WHO criteria [44]. Such a pattern is indicative of hypoxic alterations and was initially described in cerebral malaria patients from the same cohort [46], suggesting that brain involvement is not restricted to comatose patients. Because these hypoxic changes were directly proportional to plasma levels of creatinine [17], AKI in severe, noncerebral malaria may increase brain susceptibility to cytotoxic edema [17,46]. Furthermore, elevated levels of S100B, a protein biomarker that reflects central nervous system injury [47], were also reported in this patient category when compared to uncomplicated malaria controls. As increased plasma S100B levels have been linked to long-term neurocognitive repercussions in individuals with pathologies including HIV [48], small-vessel disease [49], traumatic brain injury [50] and sepsis-associated encephalopathy [51], these results suggest that neurological sequelae may be more frequent than initially thought in noncomatose adults with AKI.

In addition to a strong association with AKI, tau, a marker of injury to neuronal axon terminals, is elevated in circulation and correlated with AKI stages using the KDIGO criteria, as well as with markers of endothelial/cellular dysfunction (soluble vascular cell adhesion molecule 1, E-selectin, P-selectin, and angiopoietin 1) in both pediatric cerebral malaria and severe malarial anemia [52,53]. Ubiquitin carboxy-terminal hydrolase L1 (UCH-L1) and neurofilament light chain (NfL) are additional central-nervous-system-specific markers indicative of neuronal damage and long-myelinated axon disruption, respectively, that are elevated and correlate with AKI. Of these, tau appears to be the strongest predictor of persisting neurologic deficits and impairments in overall cognition, attention, and memory over 24 months post-cerebral malaria [52]. However, significant elevations in levels of these brain-specific markers of injury in children with severe malaria compared to asymptomatic community children, and the association with AKI and endothelial/cellular dysfunction suggests that AKI may contribute to BBB disruption and acute brain injury, with likely long-term effects. While renal dysfunction or expression by other organs and cell types cannot be ruled out as alternative sources for these markers, they are predominantly expressed by neurons and are established predictors of central nervous system injury [54]. Further, significant correlations between cerebrospinal fluid and plasma levels of tau in matched samples from pediatric cerebral malaria cases [52] suggest that, in addition to migration across a disrupted BBB, **glymphatic** clearance is more likely rather than extracerebral sources [55]. Lastly, UCH-L1 and NfL concentrations in patients with cerebral malaria and AKI were significantly higher compared to children with cerebral malaria only, with a steady increase in biomarker concentrations by stage of AKI.

## Neuroimaging identifies common susceptible brain areas in cerebral malaria and kidney disease

Neuroimaging has allowed the identification of brain regions affected in patients with kidney disease, with most studies performed in adults [56]. Vasogenic edema often occurs in the basal ganglia and its adjacent white matter in patients with kidney failure, usually in conjunction with chronic diabetes and thus prior vascular damage, as well as with metabolic acidosis [57]. Vasogenic edema in the cortical and subcortical regions is also common in these patients, [57]. In most cases, the parieto-occipital lobes are affected, showing imaging features of PRES, which has several risk factors, including kidney failure [58]. Remarkably, in children with early CKD without kidney failure, an increase in gray matter volume can be detected by quantitative volumetry, indicating that early CKD without kidney failure may also lead to vasogenic edema of this brain region [59]. Additionally, the involvement of the supratentorial white matter is frequent in both adult and pediatric CKD patients without kidney failure [60,61]. CKD has been identified as a risk factor for small-vessel disease, leading to white-matter lesions, lacunar infarcts, and subcortical microhemorrhages [62–64]. Impaired cerebral autoregulation, remodeling of the cerebral vasculature, inflammation and endothelial dysfunction could explain the additive mechanisms through which renal dysfunction leads to cerebral small-vessel disease [65]. On MRI, altered cerebral blood flow indicative of decreased cerebrovascular autoregulation correlates with neurocognitive symptoms in children and young adults with CKD [66]. Overall, kidney function decrease is associated with neurocognitive decline, highlighting the impact of kidney dysfunction on brain alterations [63,67]. Taken together, these findings illustrate that kidney dysfunction causes brain pathology identifiable by neuroimaging, and a kidney–brain axis influencing brain cortical structures was recently evidenced by a Mendelian randomization study [68]. Interestingly, similar brain regions are affected in patients with kidney failure and cerebral malaria. Indeed, both diseases have been associated with PRES imaging features with predominant brain swelling in the parieto-occipital lobes [34,69], although there is a stronger cytotoxic edema component in cerebral malaria patients compared to AKI and CKD patients with kidney failure [70]. In addition, subcortical microhemorrhages are frequent in both CKD and cerebral malaria [63,71], a feature that extends to the basal ganglia, corpus callosum, and cerebellum in the latter [71]. Even though the contribution of other underlying causes cannot be excluded, it is remarkable that kidney and brain function are closely interconnected, and an inflammatory crosstalk is highly likely between the two organs [28]. The described imaging findings further emphasize pathogenic similarities between both conditions, with small vessels being affected and resulting in lesions in similar brain areas. In view of the recent findings from Uganda and India, it is plausible that specific brain areas are susceptible in kidney disease and cerebral malaria, leading to an exacerbation of brain involvement in patients with both. Further studies are warranted to elucidate if and how imaging findings differ between cerebral malaria patients with or without kidney injury. Stringent comparative assessment of biochemical derangements in patients with **uremic encephalopathy** compared to those in patients with cerebral malaria and/or malaria associated AKI should also be prioritized to better understand the potential respective contributions of these pathologies on brain involvement during severe falciparum malaria.

## New challenges

Taken together, these studies shed a new light on the emerging, complex, and potentially pivotal role of the kidney–brain pathogenic crosstalk in severe falciparum malaria. But this raises additional questions, notably on the accuracy of the AKI and CKD diagnosis, the current criteria used to define cerebral malaria, and on the involvement of similar mechanisms in nonfalciparum malaria, albeit to a lower extent.

**Figure 2.**
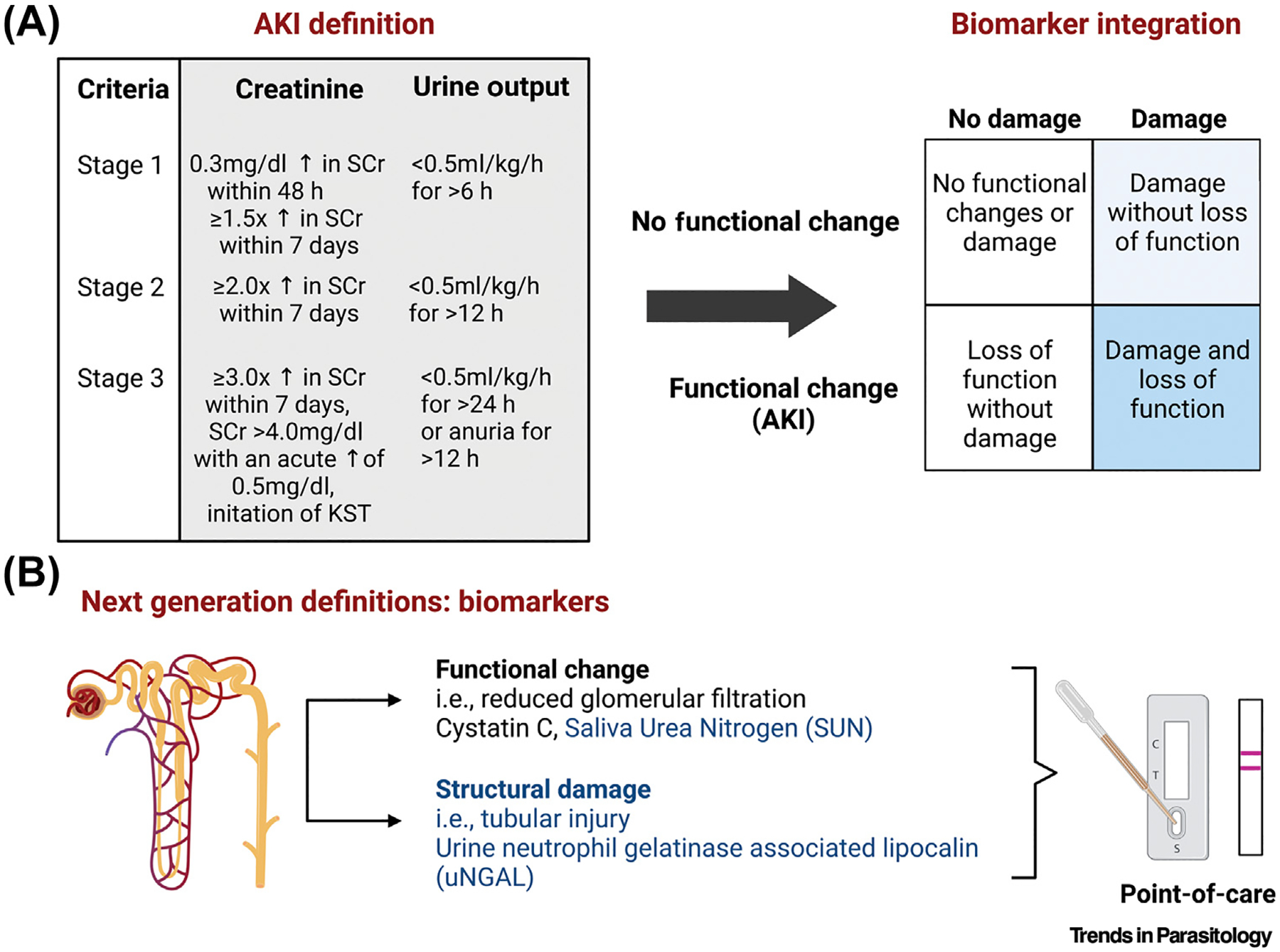
Diagnosis of acute kidney injury (AKI) using consensus criteria. AKI is defined based on an abrupt change in kidney function based on increases in serum creatinine as a marker of kidney filtration or decreases in urine output and staged based on the severity of the functional impairment. (A) Next-generation definitions integrate additional biomarkers to differentiate between functional changes in kidney filtration and structural injury/damage. (B) In addition, potential candidate biomarkers for malaria-endemic areas should be adaptable to point-of-care tests to facilitate risk stratification at the bedside. Figure created using BioRender. Abbreviation: SCr, serum creatinine.

The KDIGO group currently recommends a definition of AKI based on functional changes in the glomerular filtration rate assessed through increases in serum creatinine or decreases in urine output [37]. New proposed definitions integrate biomarkers of structural injury to the kidney which may be able to differentiate more accurately AKI and CKD in populations without known kidney function prior to infection (e.g., urine neutrophil gelatinase-associated lipocalin (NGAL), also known as lipocalin-2 [72]). While these new biomarkers undoubtedly have potential to improve recognition and awareness of AKI in the context of severe malaria, public–private partnerships will be needed to make diagnostics accessible and affordable in malaria-endemic areas. Improved recognition and awareness of AKI as a clinical complication associated with both short- and long-term morbidity and mortality and expanded access to diagnostic tools, including urinalysis, creatinine, and novel biomarkers will support early diagnosis, such as cystatin C [73], saliva urea nitrogen, urine NGAL/lipocalin-2 [74], or miRNAs [75] (Figure 2). AKI-focused supportive care, including appropriate fluid resuscitation and assessment of fluid balance, removal of non-essential nephrotoxic medications, and expanded access to kidney-replacement therapy, are needed to reduce AKI-related mortality in the context of malaria. Adjunctive therapies for severe malaria should focus on developing kidney-protective therapies that promote acute, but also long-term, recovery. One kidney protective strategy is regularly dosed acetaminophen to address kidney injury associated with lipid peroxidation from cell-free hemoglobin, with promising trials in Asian adults with *P. falciparum* (NCT01641289) and *Plasmodium knowlesi* (NCT03056391), and an ongoing study in African children with *P. falciparum* (NCT04251351) [76,77]. Additional studies are needed to understand the pathobiology of AKI and pathways associated with kidney recovery to promote long-term recovery and, potentially, improve neurocognitive outcome.

Similarly, the current definition of cerebral malaria using a Glasgow Coma Score (GCS) of <11 in adults is inadequate to identify patients with brain involvement [17]. Indeed, patterns of cerebral hypoxic injuries identical to the ones seen in cerebral malaria were revealed by MRI in severe, noncerebral malaria adult patients with GCS ranging from 12 to 14, and mild vasogenic edema was commonly seen in uncomplicated malaria cases [17]. While reversible upon treatments, these transient changes may have long-lasting effects post-recovery and need to be investigated. To facilitate this, alternative diagnostic approaches to circumvent the logistical and financial challenges associated with neuroimaging are necessary and could include promising circulating biomarkers such as NGAL/lipocalin-2 or micro-RNAs miR-150–5p and miR-3158–3p [46,78].

Lastly, the prevalence of kidney dysfunction in nonfalciparum malaria and its potential effects on the brain are currently unknown. AKI is frequent in severe *Plasmodium vivax* [79–82] and *P. knowlesi* infection [83], and has been described in patients with *Plasmodium malariae* [84] and *Plasmodium ovale* [85], albeit anecdotally. While underlying pathogenic pathways may differ depending on the malaria species, additional studies are needed to understand whether AKI may relate to brain injury in multiple species. The implementation of the KDIGO criteria across all malaria cases to assess AKI, coupled to the measurement of circulating brain injury biomarkers, could reveal the extent of kidney–brain crosstalk effects in nonfalciparum species. It is noteworthy that the incidence of CKD following AKI has never been assessed in these patient categories. The prevalence of CKD is of ~15% in India [86], and could be linked to the country’s high *P. vivax* endemicity.

## Concluding remarks

The recent emergence of a pathogenic role for the kidney–brain crosstalk during severe falciparum malaria should be urgently taken into consideration to reassess diagnostic approaches for both kidney and brain dysfunction in severe disease. This effort needs to be expanded to all malaria parasite species, to identify pathogenic pathways and inform new adjunctive therapies, but also to detect patients at risk of developing long-term neurodisabilities and develop rehabilitation protocols. The potential clinical benefits of kidney protective strategies, such as acetaminophen, have been recently demonstrated in adults with severe malaria. However, the impact of such treatment on the subsequent development of CKD, and its possible neuroprotective effects, need to be carefully evaluated during follow-up studies (see Outstanding questions). In parallel, mechanistic studies using animal or *in vitro* models are warranted to elucidate the mechanisms underlying this potential kidney–brain crosstalk in falciparum malaria. Meanwhile, new evidence shows the importance of the very promising ‘blanket’ protection against both kidney and brain damage afforded by malaria chemoprevention [12], which should be scaled up where possible.

## Highlights

New evidence highlights a potential role for kidney injury in the development of neurocognitive sequelae in children with severe falciparum malaria.

In parallel, neuroimaging studies have demonstrated that adults with severe malaria and acute kidney injury have brain changes identical to the ones seen in cerebral malaria.

This emerging kidney–brain pathogenic axis is novel in the context of severe malaria and, to investigate its long-term effects rigorously, the definitions of acute kidney injury and cerebral malaria need to be reconsidered.

The use of biomarkers may be pivotal to identify patients with altered kidney and brain function, thereby informing their clinical management and rehabilitation.

In the meanwhile, kidney protective therapies and malaria chemoprevention may help to decrease malaria-associated neurodisabilities.

## Outstanding questions

What is the extent of neurocognitive sequelae in adults who recover from cerebral malaria? Are these transient or long term? They remain to be investigated with rigorous, long-term follow-up assessments. Similarly, adults recovering from AKI in the context of severe malaria and without apparent brain involvement should also be evaluated urgently.

To facilitate the above, can urine or plasma biomarkers be deployed for the detection of brain and/or kidney involvement in severe malaria cases? Combined with more stringent clinical criteria, could these provide a higher diagnostic and prognostic granularity for patients to inform their clinical and recovery management?

Does a similar brain–kidney pathogenic axis exist in nonfalciparum malaria? Can biomarkers also help the assessment of AKI and potential silent cerebral malaria in *P. vivax*, in particular?

Can kidney-protective strategies contribute to alleviate the detrimental effect of AKI on the brain in severe malaria?

## Glossary

Acute kidney injury (AKI): a sudden episode of kidney damage or failure that develops in hours or days and causes a build-up of toxic waste products in the blood
Cytoadhere: binding of infected erythrocytes to endothelial cells in different organs as a mechanism to evade host immune removal, leading to sequestration
Endothelium: a thin cell monolayer that lines blood vessels and releases substances controlling vascular contraction and relaxation, blood clotting, and immune functions
Excitotoxic: a loss of neuronal function and cell death resulting from the toxic actions of excitatory amino acids
Glycocalyx: a dense, gel-like meshwork that surrounds some cells, constituting a physical protective barrier
Glymphatic (system): a recently discovered macroscopic waste clearance system that utilizes perivascular channels formed by astroglial cells to eliminate soluble proteins and metabolites from the central nervous system
Hippocampus: a complex brain structure embedded in the temporal lobe that plays a vital role in regulating learning, memory encoding, memory consolidation, and spatial navigation
Neurodisability: an umbrella term for conditions associated with impairment of the central nervous system, including conditions such as cerebral palsy, autism, and epilepsy; these can co-occur
Sequelae: complications or conditions that result from a pre-existing illness, injury, or trauma
Uremic encephalopathy: an uncommon metabolic disorder syndrome characterized by reversible neurological symptoms in patients with acute kidney injury or severe chronic kidney disease; it may result from multiple metabolic derangements
